# Mesoporous Silica Nanoparticles as Drug Delivery Vehicles in Cancer

**DOI:** 10.3390/nano7070189

**Published:** 2017-07-22

**Authors:** Anna Watermann, Juergen Brieger

**Affiliations:** Department of Otorhinolaryngology, Head and Neck Surgery, University Medical Center Mainz, Langenbeckstraße 1, 55131 Mainz, Germany; awaterma@uni-mainz.de

**Keywords:** mesoporous silica nanoparticles, drug delivery, tumor targeting, biocompatibility

## Abstract

Even though cancer treatment has improved over the recent decades, still more specific and effective treatment concepts are mandatory. Surgical removal is not always possible, metastases are challenging and chemo- and radiotherapy can not only have severe side-effects but also resistances may occur. To cope with these challenges more efficient therapies with fewer side-effects are required. One promising approach is the use of drug delivery vehicles. Here, mesoporous silica nanoparticles (MSN) are discussed as biodegradable drug carrier to improve efficacy and reduce side-effects. MSN excellently fulfill the criteria for nanoparticulate carriers: their distinct structure allows high loading capacity and a plethora of surface modifications. MSN synthesis permits fine-tuning of particle and pore sizes. Moreover, drug release can be tailored through various gatekeeper systems which are for example pH-sensitive or redox-sensitive. Furthermore, MSN can either enter tumors passively by the enhanced permeability and retention effect or can be actively targeted by various ligands. PEGylation prolongs circulation time and availability. A huge advantage of MSN is their explicitly low toxic profile in vivo. Yet, clinical translation remains challenging. Overall, mesoporous silica nanoparticles are a promising tool for innovative, more efficient and safer cancer therapies.

## 1. Introduction

Although cancer therapy has improved over the past decades and survival rates increased [1], the heterogeneity of cancer still demands new therapeutic strategies. Especially solid tumors at anatomical crucial sites e.g., glioblastoma, head and neck squamous cell carcinoma, lung adenocarcinoma are sometimes limited to radiotherapy and/or chemotherapy. Nonetheless, detrimental effects of these therapies are chemo- and radioresistance which promote locoregional recurrences, distant metastases and second primary tumors. Besides, severe side-effects reduce the patients’ quality of life. Therefore, it is of utmost importance to develop new therapeutic strategies to overcome resistances and to reduce side-effects by targeted therapy. One possibility is to embrace the enhanced permeability and retention (EPR) effect of solid tumors: Due to a leaky vasculature and the lack of lymphatic drainage small structures such as nanoparticles can accumulate in the tumor [2]. Therefore, exploiting nanoparticles as drug delivery vehicles is a promising approach. 

Research in nanomedicine prospered over the last decades and yielded several prerequisites for drug delivery systems. Nanoparticles should have a high loading capacity and the cargo should be protected until it reaches the side of action. Moreover, nanoparticles should be taken up predominantly and efficiently by cancer cells and evade the mononuclear phagocytic system (MPS). Once drug carriers are incorporated by the cells, endosomal escape and drug release is crucial. Good tumor accumulation and deep tumor penetration are also favorable. Importantly, nanoparticles need a good biocompatibility which is dependent on the used material but also influenced by degradation and excretion.

Over the past decades a plethora of different nanoparticles for drug delivery, organic and inorganic, were developed. Organic nanoparticles are represented for example by liposomes, polymer micelles, dendrimers and poly lactid-co-glycolic acid (PLGA)-based nanoparticles. In fact, some liposomal formulations are already approved by the US Food and Drug Administration (FDA), e.g., liposomal doxorubicin (Doxil^®^/Caelyx™) for treatment of Karposi’s sarcoma, ovarian cancer and multiple myeloma [3]. Yet, the advantage of liposomes compared to the free drug is mostly limited to longer half-life and reduced toxicity [4]. Furthermore, several polymeric and micelle based vehicles for cancer therapy were or are in clinical trials, respectively [3]. 

Drug delivery systems can also be based on inorganic materials, e.g., gold nanoparticles, metal oxide such as iron oxide particles, carbon nanotubes, quantum dots and mesoporous silica nanoparticles (MSN) [5,6,7,8,9]. Particularly, iron oxide nanoparticles are already approved for glioblastoma therapy in Europe and as contrast enhancers for magnetic resonance imaging [3]. So far, no clinical trials were performed with MSN but an early phase I study (NCT02106598) is conducted with targeted silica nanoparticles for image-guided operative sentinel lymph node mapping [10]. However, MSN exhibit several superior features in comparison to other inorganic nanoparticles: MSN possess a unique structure with a tunable pore and particle size, resulting in a high specific surface area which can be easily functionalized, and most importantly are highly biocompatible. Silica is classified as “Generally Recognized as Safe” (GRAS) by the FDA and is used in cosmetics and as a food-additive [11]. The MSNs’ porous structure allows a high drug loading capacity and a time-dependent drug release. But, the cargo can also be absorbed to the particle’s surface. The pores are usually sealed by a gatekeeper system which is often also used for additional functionalization and improvement of pharmacodynamical characteristics.

In the following paragraphs we will discuss MSN synthesis, characteristics and surface modifications with regard to cancer cell targeting, controlled drug release and endosomal escape. Finally, MSN biocompatibility in vitro and in vivo will be reviewed and challenges of MSN application in cancer therapy will be discussed.

## 2. MSN Synthesis and Characteristics

First, MSN synthesis will be discussed briefly with regard to nanoparticle diameter and pore size. Then, the influence of the nanoparticles’ characteristics is described with regard to drug delivery vehicles.

### 2.1. MSN Synthesis

Several different approaches are used for mesoporous silica nanoparticle synthesis resulting in a variety of engineered particle and pore sizes. For instance, MSN are synthesized based on a modified Stöber synthesis, using e.g., tetraethyl orthosilicate (TEOS) as precursor for silica condensation and different additives as templates such as surfactants like cetyltrimethylammonium bromide (CTAB), polymers, micelle forming agents or other dopants [12,13]. In brief, surfactants are stirred in a mixture of water and alcohol under basic conditions and TEOS or other silicates are added under agitation. Concentrations and compositions of silica sources, template-agents and stirring conditions determine particle size, pore size and shape. When the surfactant concentration is above the critical micelle concentration, CTAB self-aggregates into micelles and the silica precursor condensates at the surface. A silica structure is formed around the surface of the micelles. Then, the surfactants have to be completely removed to obtain biocompatible mesoporous silica nanoparticles which are usually further modified [14]. Another approach was first introduced by Zhao et al. who used triblock copolymers as templating agents for well-ordered hexagonal mesoporous silica structures with up to 30 nm pores [15]. The common pore size of MSN ranges between 2 and 5 nm but larger pore sizes of 23 nm can be generated e.g., by adding swelling agents such as trimethylbenzene [16]. Also, hollow-structured MSN were examined as drug carriers by Wu et al. who employed a stability difference-based selective bond breakage strategy. In brief, this strategy relies on the fact that a Si–C bond is weaker than a Si–O bond and can be degraded by hydrothermal treatment. By applying different temperatures, pore sizes were increased gradually up to 24 nm [17]. A greater variation can be found in the particle diameter which is also dependent on surface modifications. While some silica nanoparticles are 100–120 nm in diameter others are larger than 200 nm, yet pore sizes are similar (2.5 nm or 3.0 nm, respectively) [18,19,20]. However, the denoted particle diameter is also dependent on surface modifications such as coatings and the suspension medium. A more detailed description of MSN synthesis strategies can be found here [14].

### 2.2. Influence of MSN Characteristics on Biological Systems

The influence of nanoparticle characteristics including size, shape, surface area and chemistry on biological systems play an important role for efficient drug delivery and was extensively reviewed by Albanese, Tang and Chan [21]. MSN exhibit a high specific surface area of up to 1000 m^2^/g which is decreased by surface modifications such as amination or coating [22,23]. Accordingly, large-pore nanoparticles (10 nm) exhibit a smaller specific surface area [24]. Yet, a large surface area increases loading efficiency for small molecule drugs and siRNA. For example, a nearly 1000 fold higher amount of Doxorubicin could be loaded in MSN compared to the FDA-approved liposomal formulation Doxil^®^ [25]. MSN uniformity is important for quality assurance and can be determined by dynamic light scattering. Analyzing the Brownian motion reveals the polydispersity index (PDI) as indication for the colloidal dispersion size range and a low PDI is favorable [26]. Also, particle shape and size are analyzed with transmission electron microscopy (TEM). The nanoparticle characteristics such as shape, size and charge have an influence on particle uptake. Cellular entry is also dependent on the applied targeting strategy. In general, several possible endocytic pathways for cellular nanoparticle uptake were proposed, namely caveolae or clathrin-dependent endocytosis, caveolae or clathrin-independent endocytosis and micropinocytosis [27]. The most prominent cellular entry strategy is receptor-mediated endocytosis of targeted nanoparticles. After the MSNs’ ligands bound the corresponding receptors on the cellular membrane, the endocytic process is initiated and particles are incorporated in endosomes [28]. Yet, untargeted MSN can also interact with the plasma membrane through their surface modifications by non-specific binding forces and then are endocytosed or penetrate the cellular membrane [29]. Regarding the nanoparticle shape, the best cellular uptake was achieved by rods, followed by spheres, cylinders and cubes when particles were larger than 100 nm [21,30]. Yet, spherical MSN of 50 nm showed a notable better incorporation by HeLa cells than 110, 170 or 280 nm particles, respectively [31]. The membrane-wrapping process and ligand-receptor interactions influence the uptake efficiency of different particle sizes. A smaller nanoparticle of 50 nm is able to induce membrane-wrapping by binding a sufficient number of receptors. While larger nanoparticles interact with a higher number of receptors and the uptake is limited by the receptors’ redistribution on the cellular membrane through diffusion to compensate for local receptor shortage [21,29]. Since endosomes exhibit an acidic pH and pH decreases along the endocytic pathway from late endosomes to lysosomes [32], this acidic environment is used for a controlled release strategy, which is reviewed later. The surface charge also influences nanoparticle uptake. Positively charged particles have been found to be taken up faster than neutral or negatively charged particles by human cancer cells [28]. The cellular membrane has a slightly negative charge and favors binding of positively charged nanoparticles by electrostatic interaction. Yet, in a physiological environment nanoparticles are coated by a protein corona consisting of serum proteins, opsonins and ions which changes the in vitro determined parameters such as size and charge and thereby also influences cellular uptake and toxicity [29]. The absorbed proteins facilitate clearance by the MPS and agglomeration, but this can be prevented by coating the nanoparticles with poly(ethylene glycol) (PEG) resulting also in longer blood-circulation times [16,33]. So, MSN size and shape have a great influence on the nanoparticles’ in vitro and in vivo behavior. Yet, surface modifications have an even greater impact on the drug delivery vehicles properties and will be discussed next.

## 3. Modifications to Control Cellular Uptake, Drug Release and Endosomal Escape

MSN surface modifications are necessary for several purposes: targeting moieties are supposed to direct the drug carrier to the desired destination, different capping systems ensure controlled drug release at the site of action and endosomal escape is not only crucial for efficacy but can also be influenced by certain alterations. The silanol groups present within the interior of the pores and on the outer surface can be modified with various functional molecules. These alterations will be discussed in the following sections.

### 3.1. Passive and Active Targeting of MSNs

Scientists imagine a site-directed cancer therapy to lower toxic side-effects, enhance efficacy and reduce required drug doses. In general, three different strategies are exploited for this purpose, namely passive targeting, active targeting and magnetic-field directed targeting. These approaches were profoundly reviewed by Yang and Yu in 2015 [34], thus only an up to date summary will be given here.

#### 3.1.1. Passive Targeting

As mentioned above, nanoparticles accumulate favorably in solid tumor tissue due to the EPR effect, which is considered as passive targeting. Generally, tumors exceeding about one cubic millimeter in size require oxygen and nutrient supply to proliferate further [35]. Therefore, they rapidly form a highly abnormal vasculature by angiogenesis. The blood vessels are lined by a single, thin layer of flattened endothelial cells, the basement membranes have fenestrations varying in size and little or no pericytes cover the vessels [36]. Hence, macromolecules larger than 40 kDa, which is the threshold of renal clearance, can leave the blood vessels and accumulate in the adjacent tumor tissue but not in normal tissue. Also, solid tumors commonly lack effective lymphatic drainage, so accumulated macromolecules or nanoparticles remain longer in the tumor tissue without being cleared by the immune system [37]. To achieve efficient passive targeting, so far the focus laid on prolonging circulation time which is dependent on renal clearance and MPS escape. Phagocytic cells such as monocytes and macrophages are mainly located in liver, spleen, bone marrow and lymph nodes [38]. Hence, nanoparticles also tend to accumulate in these organs.

For efficient passive targeting several nanoparticle characteristics have to be considered such as particle size, morphology and surface modifications. To avoid renal clearance particles have to be at least 10 nm in diameter and a size of 100–200 nm seems to be optimal to also evade the MPS [34]. Besides, the nanoparticle shape also plays a role in passive targeting based on the EPR effect and was examined by Huang et al. in vivo. Using short-rod and long-rod MSN the main accumulation was found in liver, spleen and lung, which is no surprise considering the high blood flow rate of these organs. Yet, short-rod MSN tended to preferably accumulate in the liver with a fast clearance rate while long-rod MSN were distributed in the spleen with relatively slow clearance [39]. However, this study was performed without tumors and therefore no passive targeting was shown. Lu and colleagues could demonstrate enhanced tumor accumulation of MSN in comparison to normal tissue in vivo while MSN also exhibited good biocompatibility [40].

Surface modifications also have a major influence on nanoparticle tumor accumulation. As mentioned above, PEG is used to minimize opsonization and thereby evade the MPS. However, it has been implicated that PEGylation reduces cellular nanoparticle uptake in cancer cells but also in macrophages [33,41,42]. Nevertheless, Zhu and colleagues reported improved uptake of PEGylated hollow MSN in comparison to naked particles in cervical cancer cells and mouse embryonic fibroblasts [43]. Another considerable aspect with regard to passive targeting is the elevated interstitial fluid pressure in solid tumors which can be 10 to 40 fold higher compared to normal tissue [44]. This can create pressure gradients and heterogeneous flow in the interstitium which influences the distribution of nanoparticles and can lead to reduced particle concentrations in the tumor. Nonetheless, larger tumors and metastases often have necrotic tissue or highly hypovascular areas in the center because angiogenesis was slower than tumor growth. For this reason, nanoparticles can barely reach these regions by passive targeting.

Moreover, based on the data collected and analyzed by Wilhelm and colleagues [36] only 0.4 ± 0.2% of the administered untargeted MSN dose (7 data sets) could be found in the tumor tissue. However, 0.8 ± 0.5% of injected targeted MSNs (6 data sets) were found in tumors supporting the advantage of active targeting which will be discussed in the following paragraphs.

#### 3.1.2. Active Targeting

**Figure 1:**
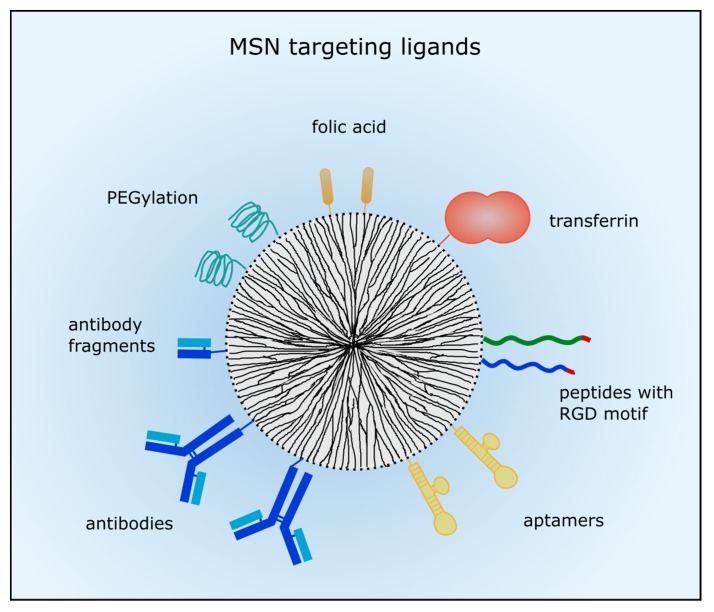
Ligands for active tumor targeting. MSN can be coated with poly (ethylene glycol) (PEG) to prolong circulation time. Small molecules such as folic acid are often used for active targeting. Different peptides with the arginine-glycine-aspartic acid (RGD) motif or proteins such as transferrin were also employed for tumor targeting. Moreover, aptamers, antibodies or antibody fragments are utilized to target membrane-receptors which are commonly overexpressed in cancer cells.

In order to enhance drug delivery with nanocarriers and drug efficacy, active targeting is conducted to membrane receptors predominantly expressed in tumors, in vascular structures or in the nuclear membrane. In case of leukemic diseases nanoparticle targeting is inevitable because the EPR effect does not apply. So, different targeting moieties can be added to the MSNs’ surface such as small molecules, short peptides, aptamers and whole antibodies or antibody fragments. Usually, the MSN are then taken up by receptor-mediated endocytosis. An overview of the described targeting ligands is given in Figure 1.

A prominently used tumor cell target is the folate receptor which is overexpressed in many tumors in comparison to healthy tissue [45]. Qi et al. targeted laryngeal carcinoma with folic acid-modified MSN. They successfully delivered commonly used chemotherapeutic drugs (paclitaxel, cisplatin, 5-fluoruracil) and siRNA targeting ABCG2, a drug efflux pump involved in multidrug-resistance, to CD133^+^ positive laryngeal cancer cells in vitro and in vivo [46]. Before, the group showed a greater reduction in laryngeal tumor size in a mouse model by using cisplatin-loaded and folate-conjugated MSN compared to untargeted MSN [19]. Zhang and colleagues also utilized folate as targeting ligand on MSN to improve the radioenhancer effect of valproic acid in glioblastoma cells [47]. Moreover, PEG-conjugated folate was applied by Cheng et al. as targeting ligand on pH-sensitive polydopamine coated MSN in vitro and in vivo. Doxorubicin delivery via folate-targeted MSN had improved efficacy compared to the free drug and untargeted MSN with doxorubicin in a xenograft tumor model. Also, distinctly higher tumor accumulation of folate-targeted MSN in comparison to untargeted nanoparticles was observed [48]. 

Using another concept, the glycoprotein transferrin was applied as targeting-ligand and redox-responsive gatekeeper by Chen et al. who could show the same toxicity of the free drug doxorubicin and doxorubicin in transferrin-targeted-MSN in hepatocellular carcinoma cells [49]. Furthermore, Chen and colleagues exploited the fucose-binding lectin UEA1 for colorectal adenocarcinoma, adenoma and polyposis coli targeting and detection. Fluorescently labeled and UEA1 carrying MSN were successfully tested in a mouse colon cancer model as a contrast agent to visualize malignant lesions in the colon [50].

Not only proteins can be utilized for targeting but also short peptides. For instance, Sweeney and coworkers attached a bladder-cancer specific peptide named Cyc6 to Gd_2_O_3_-MSN and thereby improved the detection of tumor boundaries in magnetic resonance imaging (MRI) scans in a mouse bladder cancer model [51]. The arginine-glycine-aspartic acid (RGD) motif is a prominent peptide sequence targeting integrin αvβ3 which is overexpressed in certain tumors [52,53]. Therefore, peptides including the RGD motif have been used for targeting MSN to tumors in vivo by Pan et al. who showed good tumor accumulation and efficacy of doxorubicin loaded MSN. Even better tumor accumulation and reduction in tumor size were found when the cell-penetrating and nuclear-targeting peptide TAT was also coupled to the MSN besides RGD. In addition, bare MSN accumulation in liver and spleen was distinctly greater than RGD/TAT-MSN accumulation in those organs while untargeted MSN were found only in small concentration in the tumor tissue [54]. A similar approach was conducted by Ashley and colleagues who used the peptide SP-94 and a nuclear localization signal (NLS) for cancer cell and nuclear targeting, respectively. The MSN were loaded with siRNA and different chemotherapeutic drugs and then coated with a lipid bilayer which conveyed the targeting moieties, a fusogenic peptide for endosomal escape and PEG. Doxorubicin-loaded and targeted MSN significantly decreased cellular viability of hepatocellular carcinoma cells in comparison to hepatocytes which were barely affected [25].

Apart from small molecules and peptides, aptamers can be used for tumor cell targeting. Aptamers are synthetic single-stranded DNA or RNA oligonucleotides that show high affinity and specificity toward different targets. They are polyanionic and larger than small peptides but smaller than antibodies [55]. An aptamer binding to epithelial cell adhesion molecule (EpCAM) was employed by Babaei and colleagues for hepatocellular carcinoma targeting in vitro and in vivo. They encapsulated 5-Fluorouracil in MSN with citrate-modified gold nanoparticles as gatekeeper which were PEGylated and conjugated with the EpCAM aptamer. Targeted nanoparticles showed a greater reduction of cellular viability than untargeted nanoparticles. Moreover, in vivo the system was tested as a theranostic device and profoundly better tumor accumulation was observed after Rhodamine-6G loaded-MSN injection in in vivo imaging [56]. Another receptor for aptamer targeting is nucleolin which is expressed on cancer cells. Tang et al. developed a photoresponsive drug delivery system based on graphene oxide wrapped MSN for light-mediated drug release and a conjugated nucleolin-targeting aptamer. However, in vitro no difference between targeted and untargeted doxorubicin loaded MSN on cellular viability of breast cancer cells was recognized [57].

Finally, whole antibodies or antibody-fragments are used for tumor targeting of drug delivery vehicles. For instance, antibodies already approved for cancer therapy are utilized for this purpose including cetuximab (targeting (Epidermal Growth Factor) EGF receptor), trastuzumab (targeting (Human Epidermal Growth Factor Receptor 2) HER2/neu receptor) and bevacizumab (targeting (Vascular Endothelial Growth Factor) VEGF receptor) or related antibodies with similar targets. The group of Jeffery Brinker developed a drug nanocarrier named “protocell” which consists of a MSN core for drug loading and a lipid bilayer as gatekeeper and platform for surface modifications. They availed an epithelial growth factor receptor (EGFR)-antibody for targeting leukemic cells efficiently in vitro and in vivo [58]. Moreover, Zhou and colleagues conjugated rituximab to MSN and evaluated the drug delivery vehicle in vitro and in vivo [59]. Rituximab is a chimeric monoclonal antibody targeting the CD20 antigen on B cells and is approved amongst others for B-cell non-Hodgkin lymphoma therapy [60]. In a murine xenograft lymphoma model a pronounced effect on tumor volume reduction was observed for rituximab-targeted doxorubicin-loaded MSN, while the mice constitution remained better in comparison to mice treated with free doxorubicin [59]. Furthermore, for tumor vasculature targeting anti-CD105 antibody (TRC105) has been employed by Chen et al. in a murine breast cancer model. Tumor uptake of antibody-conjugated MSN was significantly larger compared to untargeted nanoparticles but still, liver accumulation 24 h after injection was witnessed [61]. The same group also used a TRC105 antibody fragment (Fab) to target dual-labeled MSN for in vivo targeted positron emission tomography (PET) imaging/near-infrared fluorescent dye (NIRF) imaging of the tumor vasculature in a mouse model [62].

In conclusion, several strategies are available for nanoparticle targeting and some have already been employed successfully in murine models. However, high accumulation in organs such as liver, spleen, lungs and kidneys still poses a problem for application in cancer therapy and regulatory approval.

### 3.2. Systems for Controlled Drug Release

**Figure 2:**
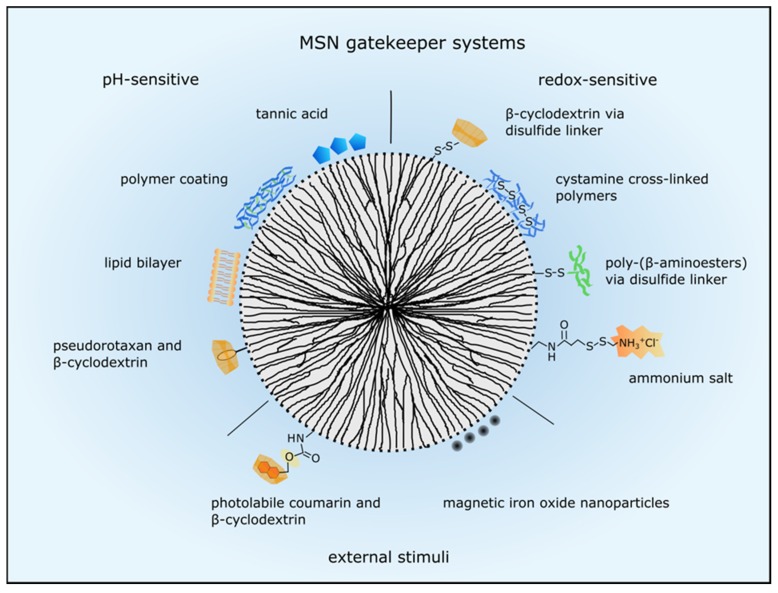
MSN gatekeeper systems to control drug release. Drug release can be regulated by internal stimuli such as pH decrease or reduction by glutathione or by external stimuli. PH-sensitive systems respond to acidic pH in the tumor microenvironment and in the endolysosomal system. Several examples are presented here such as pseudorotaxan encircled by β-cyclodextrin, tannic acid, polymer and lipid coatings. Several capping structures are linked to the MSN via disulfide bonds which are reduced by glutathione intracellularly. Then the pore blocking agents such as β-cyclodextrin, cystamine, poly-(β-aminoesters) and ammonium salt are released and the drugs can escape the nanoparticle. External stimuli such as light and magnetism are utilized to control drug release, too. Photolabile coumarin encircled by β-cyclodextrin is cleaved from the MSN by light or a magnetic field stimulates iron oxide nanoparticles to release the encapsulated drugs.

A plethora of different approaches have been used to control MSN drug release and are reviewed in detail by Mekaru, Lu and Tamanoi [63]. The various gatekeeper systems are categorized by internal and external stimuli responses and an overview of the here described examples is shown in Figure 2. Internal stimuli include decreasing pH, reducing environment and enzymes. As mentioned before, nanoparticles are often engulfed via endocytosis, so a system responding to low pH is frequently applied using different concepts. Besides, the tumor microenvironment exhibits a low pH due to hypoxia [64] and therefore, drug release can be facilitated at the target site. Examples for low pH activated capping systems include pH-sensitive nanovalves such as pseudorotaxane encircled by β-cyclodextrin [65], tannic acid [66], polymer and lipid coatings as applied by Popat et al. and Durfee et al., respectively [58,67]. Another pH-sensitive system consisted of a block copolymer containing positively charged artificial amino acids and oleic acid blocks, which acted simultaneously as capping and endosomal release agents [24]. Upon protonation the pore blocking agents were removed or degraded and the cargo could be released. Furthermore, Liu et al. developed a cascade pH-responsive system using the weak acidic pH of the tumor microenvironment and the acidic endolysosomal pH. First, β-cyclodextrin was conjugated to hollow MSN with a boronic-acid-catechol ester bond for sealing the pores which was degraded in the endosomes or lysosomes at pH 4.5 to 6.5. Second, PEG was grafted to adamantine via a weak pH sensitive benzoic-imine bond which was degraded at pH 6.8 and PEGylated adamantine reacted with the sealed nanoparticles via host-guest interactions. Therefore, PEG was released in the tumor microenvironment facilitating nanoparticle uptake and more efficient drug delivery [68]. Another dual-responsive drug carrier was developed by Liu and coworkers who induced drug release at high temperature and low pH. The polymer poly[(*N*-isopropylacrylamide)-*co*-(methacrylic acid)] was grafted onto MSN to seal the pores and control the diffusion of the cargo in and out of the pore channels depending on temperature and pH [19].

MSN drug release can also be modulated by a redox-sensitive system. As intracellular glutathione concentration can be up to 10 mM, disulfide bonds linking the capping system to the MSN are reduced upon entering the cytoplasm and cargo can be released [69]. For example, Kim et al. used β-cyclodextrin directly linked to the MSN with a disulfide bond to seal the pores and efficient doxorubicin toxicity in lung adenocarcinoma cells was shown [70]. Also, polymers cross-linked by cystamine were utilized to close the MSNs’ pores and the polymeric network was degraded in a reducing environment [71]. Besides, Wu et al. sealed their hollow structured MSN with poly-(β-amino-esters) via a disulfide-linker which was also reduced intracellularly [17]. Furthermore, a redox- and pH-sensitive dual response system was developed by Li and colleagues who utilized ammonium salt to seal the MSNs’ pores. The ammonium salt was connected via an amide and a disulfide linker to the MSN. Hence, the disulfide bond was reduced glutathione-dependently and the amide bond was degraded at low pH upon cellular uptake [72].

Using a biomolecule activated system, Mondragón et al. encapsulated camptothecin in MSN with a protease cleavable ε-poly-l-lysine and in human cervix epitheloid carcinoma cells viability was reduced after camptothecin-loaded nanoparticle incubation [73]. The same group also magnetic several hydrolyzed starch products as saccharides for enzyme-responsive drug release [74].

Apart from internal stimuli also external stimuli such as light or magnetic fields are utilized to control gatekeepers. These systems can generate more precise and local drug release, hence reducing toxicity towards normal cells. With regard to light activated drug release, the best wavelengths for adequate tissue penetration are within the biological spectra, typically 800–1100 nm [63]. In an in vitro model, Guardado-Alvarez et al. used two-photon excitation at 800 nm to cleave the nanoparticles’ cap which consisted of photolabile coumarine-molecules bound to the nanoparticle surface and non-covalently conjugated β-cyclodextrin molecules [75]. Moreover, Croissant and colleagues also used two-photon light to control drug release via a photo-transducer from mesoporous silica nanoimpellers in human cancer cells [76]. However, tissue penetration of light is still limited, so using a magnetic field for external stimulated cargo release is more advantageous even though a magnetic component is necessary. Therefore, a magnetic iron oxide core is coated with mesoporous silica or MSN are capped with iron oxide nanoparticles [77,78]. The iron oxide core has superparamagnetic properties and can be heated up by an oscillating magnetic field which in turn can be used to open a nanovalve and for example release doxorubicin [78]. Several superparamagnetic iron oxide nanoparticles (SPION) are already FDA-approved imaging agents (endorem^®^/umirem^®^ AMAG pharmaceuticals, Waltham, MA, USA) and iron oxide nanoparticles (Nanotherm^®^, MagForce, Berlin, Germany) are also approved in the European Union for glioblastoma therapy [3].

### 3.3. Endosomal Escape of MSN and Their Cargo

**Figure 3:**
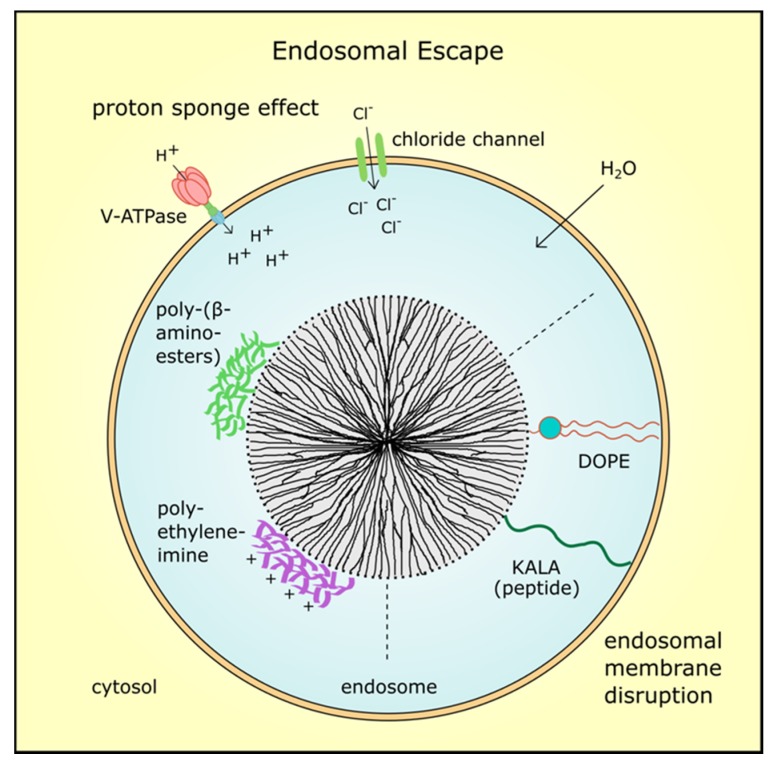
Endosomal escape mechanisms. After MSN were taken up by endocytosis, an endosomal escape is mandatory for drug efficacy. Coating with cationic polymers such as polyethyleneimine or poly-(β-aminoesters) induces the proton sponge effect. The proton concentration increases during hydrolysis which leads to an increase in membrane potential and influx of counter-ions such as chloride ions. Finally, osmotic swelling by water inflow bursts the endosome and the MSN with its cargo is delivered into the cytosol. Also, fusogenic peptides such as KALA or zwitterionic co-lipids such as dioleoyl-phosphatidylethanolamine (DOPE) can destabilize the endosomal membrane resulting in MSN release.

Once MSN entered the cancer cells by endocytosis, an endosomal escape of the nanoparticles or the delivered drug is mandatory for efficacy. The endosomal pH ranges from 6.0 to 6.5, but along the endocytic pathway acidity increases and late endosomes and lysosomes exhibit a pH from 4.5 to 5.5 [32]. Thus, the MSNs’ cargo could be degraded or inactivated by lysosomal enzymes. To avoid this, several concepts are applied to enable drug release in the cell based on different theories (Figure 3). For example, endosomal escape can be achieved by the so-called “proton sponge effect”, which relies on an increase of proton concentrations during hydrolysis. This leads to an increase in membrane potential and influx of counter-ions resulting in osmotic swelling and bursting of the endosome [79]. Hence, the cargo is released to the cytosol and can take full effect. For instance, the MSN system utilized by Wu et al. released siRNA and doxorubicin into the cytoplasm after the coating with poly-(β-aminoesters) induced endosome bursting [17]. In the same way, cationic polyethyleneimine (PEI) coating can trigger the proton sponge effect which was applied by Finlay and colleagues to deliver TWIST1 siRNA to xenograft tumors and reduce tumor burden [80].

Other methods use fusion lipids, cationic polymers or peptides to destabilize the endosomal membrane by proton absorption and acidification [81]. One example is the zwitterionic co-lipid dioleoylphosphatidyl-ethanolamine (DOPE) which was also utilized in combination with a polymer to coat MSN and improve drug release [82,83]. Moreover, Ashely et al. employed a fusogenic peptide to enhance endosomal escape of protocells in hepatocellular carcinoma [25]. Fusogenic peptides referred to as KALA were conjugated to PEI-coated MSN by Li and colleagues and were used to deliver (VEGF) targeting siRNA in a xenograft tumor model. The siRNA-loaded MSN with KALA peptides inhibited tumor proliferation significantly compared to control particles without siRNA or control siRNA, respectively [84]. However, many so far developed MSN systems relied on the proton sponge effect for endosomal escape. Aside from endocytosis, other mechanisms for nanoparticle uptake are possible, thus endosomal escape is not always necessary for drug efficacy.

Overall, surface modifications play an important role for efficient drug transport via MSN, MSN targeting and drug release. However, the “perfect” system does not exist and is unlikely to be invented due to the heterogeneity of cancer.

## 4. Biocompatibility of MSN

A major advantage of MSN is its high biocompatibility in vivo. Several studies examined biodistribution, toxicity and excretion of MSN. The FDA classified silica as “Generally Recognized as Safe” and silica is used as a food-additive and in cosmetics [11]. In general, silica particles are degraded into water-soluble orthosilicic acid (Si(OH)_4_) which is also absorbed by humans to form silica as a trace element [85]. Many in vitro studies showed no toxicity for up to 100 µg mL^−1^ MSN in cell culture [48,58,59,85]. Sometimes even higher concentrations were tested without significant toxicity [49,86]. It is generally recognized that crystalline silica nanoparticles can cause reactive oxygen species (ROS) formation which compromises cellular viability [87]. Yet, MSN seem to induce ROS formation only in high concentrations. For example, MSN concentrations of 1 mg mL^−1^ and higher exhibited ROS in colon carcinoma cells while 200 µg mL^−1^ did not induce ROS [88]. Furthermore, a relatively small MSN concentration did not promote ROS formation in hepatocellular carcinoma cells [89]. Elle and colleagues covalently coated MSN with antioxidants to reduce ROS formation and rutin decreased ROS formation dose-dependently in a keratinocyte cell line and dose-independently in colon carcinoma cells [20]. However, ROS formation after MSN application has been rarely examined due to the overall good biocompatibility.

One of the first in vivo studies was conducted by Park and colleagues who examined biodistribution of silica for four weeks. A relatively low dose of 20 mg kg^−1^ MSN (126 nm diameter) was administered intravenously into mice and the body weight increased in the same manner as in the control group. The nanoparticles predominantly accumulated in MPS-related organs such as the liver and spleen. Yet, after one week MSN were mostly cleared from the analyzed organs (liver, spleen, heart, kidney, brain and lung) and almost completely vanished after four weeks. Moreover, histopathological analysis indicated no significant toxicity compared to controls, even though apparently macrophages in the liver (Kupffer cells) were swollen one day after MSN injection. The authors assumed that MSN were degraded and then excreted via the kidneys [85]. Furthermore, He and coworkers thoroughly studied nanoparticle excretion and biodistribution in vivo. On that account, MSN and PEGylated MSN of several sizes (80 nm, 120 nm, 200 nm and 360 nm) were analyzed. Fluorescently labeled MSN were evaluated in different organs with fluorescence intensity measurements of homogenized samples at several time points after injection of 20 mg kg^−1^ nanoparticles. Most nanoparticles accumulated in spleen and liver, 30 min after injection also in the lungs, and low accumulation was detected in heart and kidneys. PEGylation reduced accumulation of larger particles in the lung and overall in the spleen 30 min after injection. However, after one month smaller particles were only observed in liver and spleen in low concentrations while 200 nm particles were also detected in even lower concentrations in heart, lung and kidneys. Regarding 360 nm MSN, the lowest concentrations were found after one month, whereas PEGylated MSN were still visible in all examined organs. Besides, nanoparticle concentration of larger particles in liver and spleen decreased over time. Blood clearance of MSN was slower for PEGylated particles and after eight hours particles were barely detectable, yet the smallest MSN had the longest blood circulation time. With regard to excretion, MSN and PEGylated MSN were mainly already excreted after 30 min and smaller particles mostly within five days. However, after one month larger particles were still detectable in urine. Histopathological evaluation showed no significant tissue toxicity and inflammation one month after injection for all particle sizes compared to controls [90]. In a study conducted by the group of Tamanoi biodistribution, biocompatibility and drug-delivery efficiency of MSN was analyzed in a xenograft tumor model. First, they determined a maximal tolerated dose of 50 mg kg^−1^ spherical MSN (100–130 nm) after intravenous injection and monitoring for ten days. Then MSN were administered intraperitoneally with the same concentration in 18 doses over two months for long-term toxicity profiling. No unusual responses or behaviors compared to controls were observed and all measured hematologic factors were within normal ranges, proposing that the treatment did not induce an inflammatory response. However, all experiments were conducted in nude mice which lacked a thymus and therefore a possible T-cell response. Good biocompatibility could also be due to the fact that more than 90% of the administered silicon concentration was excreted via feces and urine within 4 days. Moreover, in a xenograft breast cancer tumor model MSN were mainly found in tumor, lung and kidneys 24 h after tail-vein injection, while 48 h after injection the spleen exhibited increased silicon concentration. Targeting with folic acid increased tumor accumulation of nanoparticles. Furthermore, camptothecin-loaded MSN reduced tumor size faster and greater after in total 18 intraperitoneal injections over nine weeks. Also, hematology profiling suggested reduced toxicity of camptothecin-loaded MSN compared to the free drug [40]. In a more recent study, Liu et al. evaluated 120 nm hollow MSN with a pH-dependent gatekeeper system in a xenograft hepatocellular carcinoma model. The untargeted but PEGylated MSN were loaded with doxorubicin and inhibited tumor proliferation over a time period of 21 days. At the same time, the mice weight increased while mice treated with the free drug lost weight. The same tendency was observed in survival analysis where mice treated with free doxorubicin all died shortly after the treatment stopped. However, half of the mice treated with PEGylated MSN survived more than one month after the last injection until the end of the experiment. In a biodistribution study after a single injection most of the particles accumulated in liver, spleen and lung whereas PEGylated MSN exhibited less accumulation. During the first week after injection, naked particle concentrations increased in liver and spleen while PEGylated MSN also increased in lung tissue. Only low concentrations of nanocarriers were detected in heart and kidney tissues. Yet, after one month MSN concentrations were decreased as expected [68]. Zhou and colleagues utilized a relatively high concentration of 100 mg kg^−1^ rituximab-conjugated MSN for toxicity and distribution analysis in vivo. After seven MSN doses during three weeks the body weight increased correspondingly to control mice and histological analysis indicated no significant pathological lesions or damages in the major organs. Still, experiments were conducted in immunodeficient nude mice and therefor a lack of pathological damages is not surprising [59].

In brief, MSN exhibited remarkable good biocompatibility in many in vivo studies so far while tested particle concentrations increased over time. Still, accumulation of nanoparticles in MPS-related organs presents a challenge but this seemed to have no major impact on the animals’ constitution and inflammatory responses remained mild. However, most studies were performed in immunodeficient mice decreasing the chances for a severe immune response. So, more studies in rodents with intact an immune system are necessary to fully evaluate the toxic profile of MSN before clinical trials. Nevertheless, the first early phase 1 clinical trial involving targeted silica nanoparticles for image-guided operative sentinel lymph node mapping is realized [10]. In conclusion, MSN are promising drug delivery vehicles for cancer therapy from a biocompatibility perspective.

## 5. Possible Challenges of MSN Application in Cancer Therapy

As mentioned above, MSN perform well in preclinical tests, yet only one clinical trial is currently performed. As for all new drugs and medicinal formulations, regulations by the FDA or the European Medicines Agency (EMA), respectively, present comprehensible and essential hurdles: From scale up of MSN synthesis to required dosage to acceptable pharmacokinetic and pharmacodynamic profiles. 

In the case of MSN, synthesis of large amounts with consistent characteristics and quality might be challenging. Moreover, drug loading must be steady and not all drugs can be incorporated in MSN in a suitable concentration. The amount of loaded drug also influences the required nanoparticle dose. The maximal tolerated dose for unmodified MSN in a murine model was found to be 50 mg kg^−1^ and an appropriate dose for human use needs to be evaluated in phase 1 clinical trials [40]. Yet, biocompatibility and efficiency are also dependent on the modifications such as targeting ligands and gatekeeper systems. For example, rituximab-conjugated MSN were even tolerated at 100 mg kg^−1^ when intravenously injected in nude mice [59]. The nanoparticle distribution and excretion was also evaluated in murine models as mentioned above [90], but more data concerning immune response and possible side-effects, especially in an functional immunogenic environment, are needed. Also, particle accumulation in liver, spleen and other normal tissue poses a hurdle for clinical translation. Failure of the MSN system might lead to a burst drug release beyond the tumor tissue and could result in systemic toxicity, so the safety of the nanoparticle system needs to be critically evaluated. However, this is also dependent on the drug, for example siRNA would probably be degraded by nucleases and not pose a substantial threat while chemotherapeutic drugs could be harmful or even life threating. When the drug vehicles reached their site of action (tumor microenvironment or tumor cells), a controlled and efficient drug release has to be ensured. Assuming a MSN system for drug delivery fulfills the above mentioned criteria, it is most likely advantageous compared to liposomal or free drug formulations. It can be loaded with higher doses, can be targeted and drug release can be controlled. This would reduce the required dose and side-effects which are due to systemic delivery. Also, delicate drugs such as siRNA could be delivered. Upon degradation MSN are broken down into non-toxic silicic acid moieties which are easily excreted via the kidneys [85]. 

In conclusion, even though MSN provided good results in preclinical studies, clinical translation progresses slowly.

## 6. Summary

New and innovative approaches are needed to combat the heterogeneous disease cancer. Here, MSN were reviewed as drug delivery vehicles to improve efficacy and reduce side-effects. MSN ideally suit the criteria for nanoparticulate carriers since their structure allows high drug loading capacity and a plethora of surface modifications. MSN can be synthesized in different sizes with distinct pore sizes. Moreover, drug release can be finely tuned through various gatekeeper systems which are pH-sensitive or redox-sensitive, for example. PEGylation promotes escape from the MPS, so circulation time and availability are prolonged. Furthermore, MSN can either enter tumors by the EPR effect or can be actively targeted by various ligands. Another huge advantage of MSN is their biodegradability and high biocompatibility in vivo. However, clinical translation still remains a challenge and needs to be addressed. All in all, mesoporous silica nanoparticles are a promising tool for innovative cancer therapy.
